# Epidemiological Modeling of the Impact of Public Health Policies on Hepatitis C: Protocol for a Gamification Tool Targeting Microelimination

**DOI:** 10.2196/38521

**Published:** 2023-09-25

**Authors:** Ricardo Baptista-Leite, Henrique Lopes, Björn Vandewalle, Jorge Félix, Diogo Franco, Timo Clemens, Helmut Brand

**Affiliations:** 1 Department of International Health, Care and Public Health Research Institute - CAPHRI, Faculty of Health, Medicine and Life Sciences Maastricht University Maastricht Netherlands; 2 NOVA Center for Global Health - Information Management School Universidade Nova de Lisboa Lisbon Portugal; 3 Exigo Consultores Lisbon Portugal

**Keywords:** hepatitis C, modeling, public health policies, patient advocacy, mobile phone

## Abstract

**Background:**

Hepatitis C is a disease with a strong social component, as its main transmission route is via blood, making it associated with lifestyle. Therefore, it is suitable to be worked on from the perspective of public health policy, which still has a lot of room to explore and improve, contrary to diagnoses and treatments, which are already very refined and effective.

**Objective:**

An interactive gamified policy tool, designated as Let’s End HepC (LEHC), was created to understand the impact of policies related to hepatitis C on the disease’s epidemiology on a yearly basis until 2030.

**Methods:**

To this end, an innovative epidemiological model was developed, integrating Markov chains to model the natural history of the disease and adaptive conjoint analysis to reflect the degree of application of each of the 24 public health policies included in the model. This double imputation model makes it possible to assess a set of indicators such as liver transplant, incidence, and deaths year by year until 2030 in different risk groups. Populations at a higher risk were integrated into the model to understand the specific epidemiological dynamics within the total population of each country and within segments that comprise people who have received blood products, prisoners, people who inject drugs, people infected through vertical transmission, and the remaining population.

**Results:**

The model has already been applied to a group of countries, and studies in 5 of these countries have already been concluded, showing results very close to those obtained through other forms of evaluation.

**Conclusions:**

The LEHC model allows the simulation of different degrees of implementation of each policy and thus the verification of its epidemiological impact on each studied population. The gamification feature allows assessing the adequate fulfillment of the World Health Organization goals for the elimination of hepatitis C by 2030. LEHC supports health decision makers and people who practice patient advocacy in making decisions based on science, and because LEHC is democratically shared, it ends up contributing to the increase of citizenship in health.

**International Registered Report Identifier (IRRID):**

RR1-10.2196/38521

## Introduction

### Background

Hepatitis C virus (HCV) is 1 of 6 oncogenic viruses, as established by the World Health Organization (WHO) [1]. Its transmission occurs primarily through infected blood, with which one may come in contact through injection drug use, unsafe injection practices, unsafe health care, and the transfusion of unscreened blood and blood products [2]. Estimates indicate that approximately 20% to 30% of the patients initially infected manage to control the acute infection, whereas approximately 75% develop chronic hepatitis C (CHC) [1,3-5].

Hepatitis C is recognized as a major public health concern, with an estimated 58 million people having CHC infection worldwide [1]. A substantial proportion of chronically infected individuals eventually develops cirrhosis or liver cancer. Approximately 190,000 people die annually from hepatitis C–related events, mostly cirrhosis and hepatocellular carcinoma (HCC) [1].

The introduction of new direct-acting antiviral (DAA) drugs dictated a paradigm change in the treatment of hepatitis C. These new drugs are much more effective and safer than their predecessors, achieving cure rates of ≥95% [6,7]. This paradigm change enabled the contemplation of disease elimination.

In May 2016, the World Health Assembly adopted the first “Global Health Sector Strategy on Viral Hepatitis, 2016-2021” [8]. The strategy encompassed a vision of eliminating viral hepatitis as a public health problem. This is encapsulated in the global targets of reducing new viral hepatitis infections by 90% and reducing deaths due to viral hepatitis by 65% by 2030 [8]. The strategy includes raising awareness; promoting partnerships; mobilizing resources; formulating evidence-based policy and data for action; preventing transmission; and scaling up screening, care, and treatment services [8]. However, in each country, several obstacles must be overcome to accomplish the WHO’s goals, many of which are related to the implementation of health policies on hepatitis C [9-12].

The effective means to eradicate hepatitis C already exist (eg, therapeutics, national health services financing therapeutics, harm-reduction services, and hepatitis C health care services), but it is still necessary to persuade decision makers by providing robust policy-relevant information that allows the implementation of necessary measures.

An interactive gamification policy tool, designated as Let’s End HepC (LEHC), was created to help stakeholders understand how different political decisions may affect the evolution of hepatitis C in their country. The objective was to create an easy and intuitive high-level communication tool to support decision-making with the aim of formulating hepatitis C eradication policies.

Currently, the tool is fully functional in Portugal, Spain, Austria, Romania, and Bulgaria and is being implemented in other countries. The policy tool is based on an epidemiological model that allows assessing the impact of the degrees of implementation of 24 public health policies (PHPs) on the evolution of hepatitis C epidemiology over time, throughout the cure cascade. The system allows for consultation in cross-platforms, such as computers, smartphones, and tablets. Several health outcomes were defined, and it is possible to observe the impact of the degree of implementation of each policy or set of policies on these health outcomes (eg, hepatitis C incidence, hepatitis C prevalence, diagnosed individuals, individuals linked to care, individuals on treatment, cured individuals, individuals with compensated cirrhosis and decompensated cirrhosis [DC], individuals with HCC, individuals requiring liver transplants [LTs], and individuals with liver-related deaths) and consequently to eliminate hepatitis C by 2030. The model contemplates the evaluation of 5 population groups at risk and the total country’s population (the sum of all populations at risk). Some policies are specific to a particular population group, thus impacting only that specific group. Therefore, the policy tool may prove to be a useful resource to support evidence-based policy-making by the involved countries’ national health authorities.

### Hepatitis C Modeling

In the past decades, several studies have developed mathematical models, including Markov chains, related to the natural history of hepatitis C, such as the work of Salomon et al [13,14]. The focus has been the modeling of incidence and prevalence rates, transmission routes, disease burden, and other factors that could be fundamental to the determination of when elimination could be achieved in different countries. Modeling in this area gained momentum with the introduction of DAA as a new effective treatment for hepatitis C in 2014 and the WHO’s targets for the elimination of hepatitis C by 2030.

Razavi et al [15] calculated the total number of individuals infected with HCV, including those newly infected with HCV, those diagnosed with HCV infection, and those cured of HCV infection, in 2013 among 16 countries. They also assessed disease burden by forecasting the disease progression and the total number of individuals at each stage of liver disease in each of the 16 countries between 1950 and 2030. Hatzakis et al [16] developed a mathematical model focused on characterizing the population infected with HCV and deaths due to hepatitis C during the same period.

In 2015, the Polaris Observatory HCV Collaborators developed hepatitis C disease burden models for 100 countries [17], with 59 models being approved by experts in each country. Using the Markov chain component in these models, which analyzed hepatitis C prevalence and genotype distribution studies published after 2013, it was possible to calculate both disease burden and disease prevalence in each country.

In 2017, Scott et al [18] conducted research on when Iceland could achieve the WHO’s elimination targets. This research was done by developing a mathematical model with a dynamic compartmental nature and performing liver disease assessment related to hepatitis C progression. The model’s targeted indicators were injecting drug status, age, and position of the individual in the HCV cascade of cure. This model comprised the analysis of hepatitis C policy implementation in the country while identifying key targets for elimination and factors that could hinder the WHO’s objective.

Chhatwal et al [19] described the importance of components for the mathematical modeling of hepatitis C, such as demographic features, the natural history of the disease, and incidence. This was illustrated for the forecast of hepatitis C burden in the United States, with the comparison of different approaches and strategies to achieve the elimination of the disease by 2030.

Models of hepatitis C have also been developed in Catalonia and applied to PHPs focused on primary health care [20], including a model involving the national health system [21].

In 2019, Tordrup et al [22] modeled the costs of resources and interventions necessary to attain the elimination goal for viral hepatitis in 67 low-income and middle-income countries.

More recently, Razavi et al [23] used Markov chains to forecast the different outcomes related to hepatitis C incidence, prevalence, diagnosis, and treatment, among others, in 45 high-income countries in relation to their progress toward the WHO hepatitis C elimination goal.

## Methods

### Inputs

For the construction of the LEHC model, the following elements were reviewed, discussed, and validated: (1) each PHP; (2) the acceptable fluctuation ranges of the PHP impact on epidemiology; (3) the application scales on which each PHP would be measured; (4) the identification of national specificities of each country where LEHC would be implemented and necessary adaptations for the modeling process; (5) the discussion of the main sources of published or unpublished statistical data and their qualification; (6) the explanation of the adaptive conjoint analysis (ACA) and its importance as an individual response; and (7) the creation of channels of permanent communication between the national researchers, scientific partners, and National Advisory Board (NAB) and the scientific coordination team of the LEHC model.

The PHP pool used in the LEHC model was obtained through literature review and in-depth interviews with experts in hepatitis C and the reviewed articles’ authors. Three subsequent deliberation sessions of 4 hours each, followed by decision-making, were held with the Portuguese NAB on February 22, 2017, to have an in-depth discussion of the final PHPs to be included in the model. According to a cure cascade reading, a total of 24 PHPs and their application scales were selected.

First, the scientific coordination team of the LEHC model application met with the scientific partners of each country involved in the project in a first moment to discuss the PHPs. Second, there was a meeting with the NABs of each country selected by the corresponding country’s scientific partner.

In sum, the epidemiological model considers 24 PHPs and corresponding scales that may impact HCV-related health outcomes in each population group being studied and discussed by approximately 50 national experts in hepatitis C.

**Figure 1:**
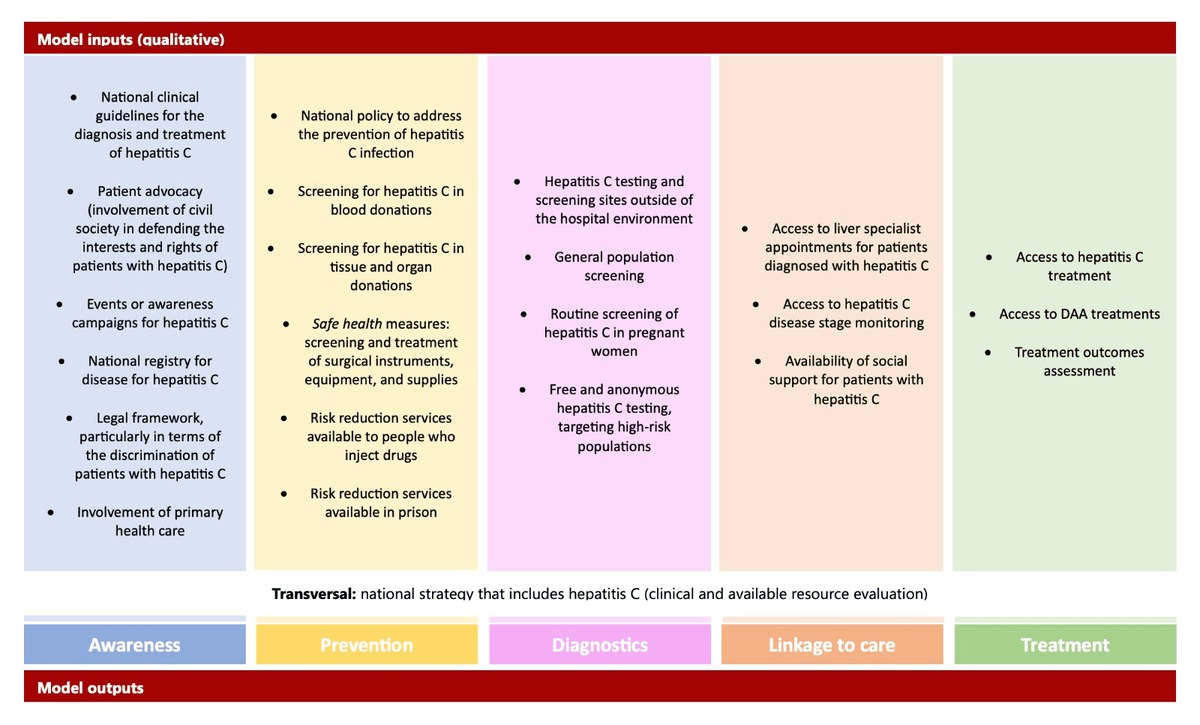
Final qualitative policies of the public health tool and respective outcomes. DAA: direct-acting antiviral.

Five groups of policies were defined based on the literature review and expert opinion (Figure 1): awareness, prevention, diagnostics, linkage to care, and treatment.

Each policy was classified into 1 of these 5 groups, thus having a direct positive impact on specific outcomes (Figure 1). Owing to their importance and relevance across the cure cascade, some policies were considered transversal, impacting all the outlined outcomes. Each policy has several possible degrees of implementation, ranging from not being available in the country to being available but not fully to being fully available. Each of these degrees results in a different impact on the model outcomes.

### Tool Outputs and Outcomes

The impact of the implementation degree of the 24 health policies can be assessed by analyzing 3 groups of indicators: hepatitis C cure, disease stages, and disease elimination within the tool.

The first group comprises the distribution of individuals throughout the cure cascade, in terms of incidence (number of new hepatitis C–positive cases), prevalence (total number of hepatitis C–positive cases), number of diagnosed individuals, number of individuals linked to specialized care, number of individuals on treatment, and number of cured individuals.

The second group comprises the distribution of individuals through the disease stages, number of individuals with compensated cirrhosis, number of individuals requiring LTs, number of individuals with HCC, and number of individuals with liver-related deaths.

The third group comprises the disease elimination (considering cutoffs of 90% in new CHC infections and 65% in mortality) [24].

These outcomes can be evaluated over time (from 2015 to 2030) for a country’s total population and each of the population segments considered in the model: (1) individuals who receive blood products, (2) prisoners, (3) people who inject drugs, and (4) individuals who are infected through vertical transmission and the remaining population (total population excluding the other 3 population groups).

### Core Model

One of the principal components of the model is an adaptation of the Markov model for the natural history of CHC infection and the impact of treatment, as published by Salomon et al [14]. This model, including stages of Metavir fibrosis and long-term complications such as compensated cirrhosis, DC, HCC, and LT, is widely adopted in the international literature on the cost-effectiveness of hepatitis C treatment and screening.

To be able to calculate the impact of different health policies on the epidemiology of CHC within a population, the aforementioned Markov model was augmented by a cascade of infection, diagnosis, and treatment (referred to as the cure cascade in the remainder of the manuscript). This cascade further divides the population into uninfected individuals, individuals who are infected but not diagnosed, individuals who are diagnosed but not retained in care, individuals who are retained in care but not treated, individuals who are treated but do not achieve subsequent sustained virologic response (SVR), and individuals who are treated and achieve subsequent SVR.

On the basis of these 2 main linked components, all individuals of a particular population subgroup (stratified by age and gender) can be represented in a set of mutually exclusive states through which individuals move over time, much in line with the auxiliary epidemiological model reported in the study by Salomon et al [14].

The impact of different policy measures on the epidemiology of hepatitis C was determined through ACA. For each of the policy measures and their implementation levels, the relative weight was determined based on the total impact on the different steps of the cure cascade.

ACA has proven to be a powerful health decision-making tool because it can go further than the traditional conjoint analysis: the computer adjusts the question following the respondent’s previous answer, instead of having hard cards; determines the relative importance of attributes; and associates the preference of choice to its importance [25], as safety procedures are set in place, defined by International Society for Pharmacoeconomics and Outcomes Research. There are 10 International Society for Pharmacoeconomics and Outcomes Research health use safety criteria [25], which were followed in this study. Multiple ACA applications have been developed in recent years in the health field: quality-adjusted life year studies [26], clinical decision-making [27], and choice of prescription [28].

### Macrolevel Description of the Conceptual Modeling Framework

#### CHC Disease Progression

**Figure 2:**
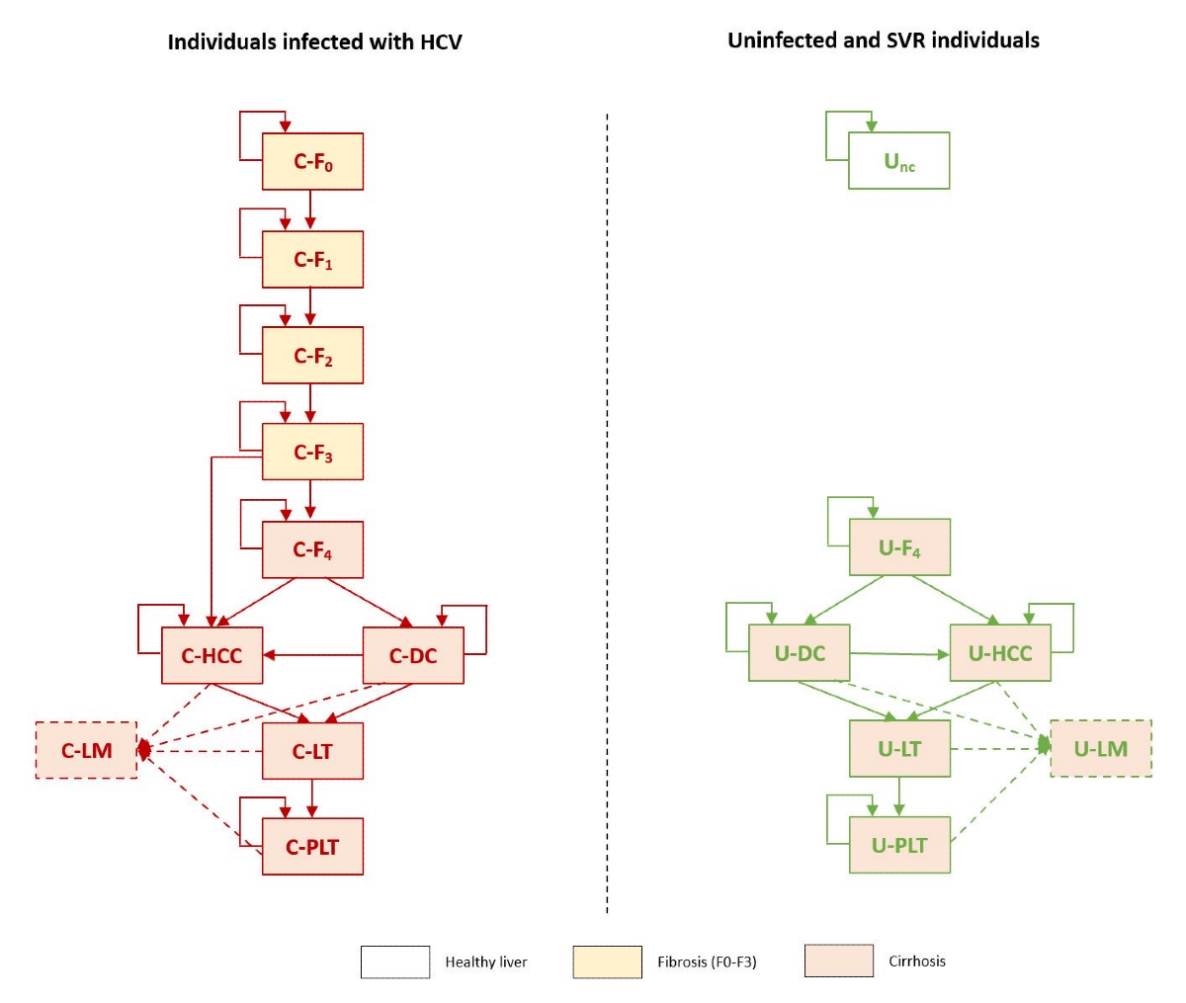
Schematic of the Markov model for the progression of chronic hepatitis C. C: chronically infected; DC: decompensated cirrhosis; HCC: hepatocellular carcinoma; HCV: hepatitis C virus; LM: liver-related mortality; LT: liver transplant; nc: noncirrhotic; PLT: post–liver transplant; SVR: sustained virologic response; U: uninfected.

In terms of CHC disease progression, the population is divided into 2 similar, but distinct, sets of health states with different transition probabilities between their respective states:

1. The first set of health states (left side of Figure 2) corresponds to individuals infected with HCV. This set represents the natural progression of CHC for untreated individuals (or individuals treated but without SVR).
2. The second set of health states (right side of Figure 2) corresponds to uninfected individuals and includes states for cirrhosis’s natural progression in individuals with SVR.

For the natural progression of CHC, mutually exclusive health states for Metavir fibrosis stages (F_0_-F_4_) and long-term complications (DC, HCC, and LT) are considered. Individuals progressing to LT pass through a tunnel state representative of the year of transplant, after which they move to a post-LT (PLT) for the remainder of their life. Possible transitions between the health states are identified by arrows (Figure 2).

The health state U_nc_ represents individuals who have never been chronically infected or attained SVR before reaching compensated cirrhosis (F_4_). Individuals who attained SVR after reaching compensated cirrhosis, although technically cleared of the virus, are considered to have had irreversible damage to the liver and face disease progression states similar to those faced by individuals chronically infected with cirrhotic (Figure 2).

No explicit health state for acute HCV infection was considered, as it was deemed not to add extra value to the model. For the same reason, no explicit health states for individuals with SVR in Metavir fibrosis stages F_0_ to F_3_ were considered. Generally presenting with reversible damage to the liver, these individuals are assumed to eventually clear fibrosis [29].

Irrespective of the health state, all individuals experience an age- and gender-dependent general mortality risk [30]. Individuals in the DC, HCC, and LT or PLTs are targeted by liver-related mortality in addition to general mortality.

The annual probabilities of transition between the different health states were obtained from the international literature and are listed in Table 1.

**Table 1:** Annual probabilities of transition between the different health states mentioned in Figure 2.

Stage from which transition occurs		Stage to which transition occurs	*P* value	Bibliographic reference
**Natural progression**				
	C^a^-F0^b^	C-F1	.12	Thein et al [31]
	C-F1	C-F2	.09	Thein et al [31]
	C-F2	C-F3	.12	Thein et al [31]
	C-F3	C-F4	.12	Thein et al [31]
	C-F3	C-HCC^c^	.008	Younossi et al [32]
	C-F4	C-DC^d^	.04	Westerhout et al [33]
	C-F4	C-HCC	.02	McEwan et al [34]
	C-DC	C-HCC	.08	McEwan et al [34]
	C-DC	C-LT^e^	.03	McEwan et al [34]
	C-HCC	C-LT	.02	Westerhout et al [33]
	C-DC	C-LM^f^	.28	Westerhout et al [33]
	C-HCC	C-LM	.51	Westerhout et al [33]
	C-LT	C-LM	.15	Westerhout et al [33]
	C-PLT^g^	C-LM	.05	Westerhout et al [33]
**With sustained virologic response (cirrhotic population only)**				
	U^h^-F4	U-DC	.005	Westerhout et al [33]
	U-F4	U-HCC	.008	Westerhout et al [33]
	U-DC	U-HCC	.04	Calculated
	U-DC	U-LT	.009	Calculated
	U-HCC	U-LT	.02	Assumed to be the same as that for C-HCC to C-LT
	U-DC	U-LM	.28	Assumed to be the same as that for C-DC to C-LM
	U-HCC	U-LM	.51	Assumed to be the same as that for C-HCC to C-LM
	U-LT	U-LM	.15	Assumed to be the same as that for C-LT to C-LM
	U-PLT	U-LM	.05	Assumed to be the same as for that C-PLT to C-LM

^a^C: chronically infected.

^b^F0-F4: Metavir stages.

^c^HCC: hepatocellular carcinoma.

^d^DC: decompensated cirrhosis.

^e^LT: liver transplant.

^f^LM: liver-related mortality.

^g^PLT: post–liver transplant.

^h^U: uninfected.

It should be noted that for individuals with SVR, the probabilities of transition from HCC to LT and from DC, HCC, LT, and PLT to liver-related mortality were assumed to be the same as those for the natural progression of the disease because no evidence was found to contradict the natural history of hepatitis C by Salomon et al [13,14].

However, scientific evidence suggests that the transition probabilities from DC to HCC and from DC to LT reduce after achieving SVR. Therefore, these transition probabilities were calculated by applying the same ratio found between the transition probabilities of the corresponding stages for patients without SVR [35].

#### Cure Cascade

Concerning the cure cascade, the population was further divided into individuals who are uninfected, individuals who are chronically infected but not diagnosed, individuals who are diagnosed but not retained in care, individuals who are retained in care but not treated, individuals who are treated but do not achieve subsequent SVR, and individuals who are treated and achieve subsequent SVR (ie, uninfected). For the ease of modeling retreatment, the key to distinguishing the pre-DAA era from the post-DAA era, individuals without SVR after the first course of treatment are eligible for a second course of treatment.

**Figure 3:**
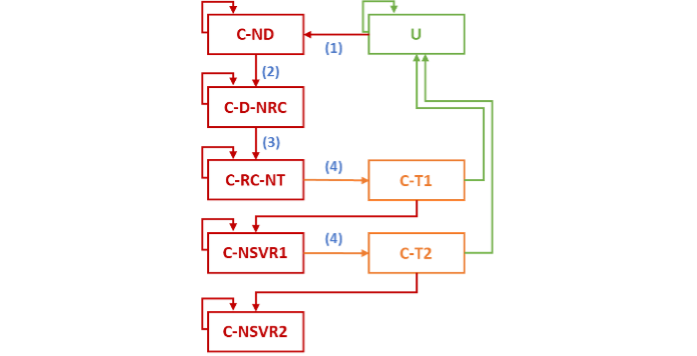
Schematic of the Markov model for the cure cascade of chronic hepatitis C. C: chronically infected; D: diagnosed; ND: not diagnosed; NRC: not retained in care; NSVR1: no sustained virologic response after first treatment course; NSVR2: no sustained virologic response after second treatment course; NT: not treated; RC: retained in care; SVR: sustained virologic response; T1: first treatment course; T2: second treatment course; U: uninfected (including individuals with sustained virologic response); 1: annual incidence; 2: annual probability of diagnosis; 3: annual probability of being retained in care; 4: annual probability of treatment.

In Figure 3, possible transitions between the different states of the cure cascade are identified by arrows.

The distribution of individuals across the different states of the cure cascade is highly dependent on the annual incidence of CHC infection (transition 1 in Figure 3), the annual probability of being diagnosed (transition 2), the annual probability of being retained in care (transition 3), and the annual probability of being treated (transition 4). These probabilities are considered variable from country to country or from population subgroup to population subgroup and are impacted by the modeled policy measures.

#### Impact of Policy Measures

**Figure 4:**
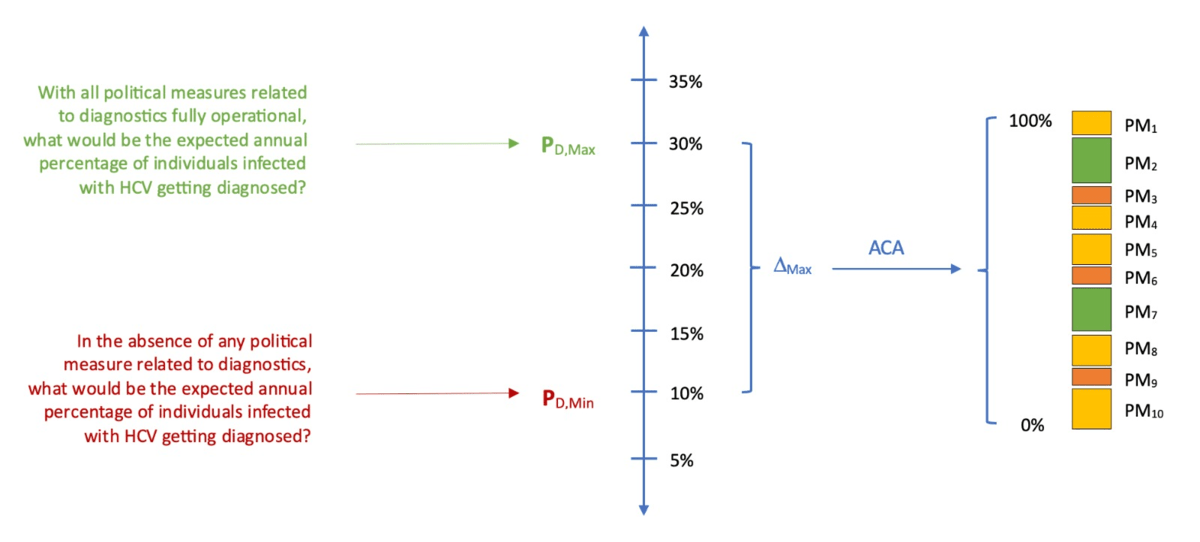
Conceptual model behind the quantification of individual policy measures’ impact on the cure cascade (exemplified with the annual probability of diagnosis). ACA: adaptive conjoint analysis; HCV: hepatitis C virus; PMi–i^th^ policy measure.

The policy measures considered in the LEHC health policy tool are of a qualitative nature. Their impact on the CHC cure cascade was quantified via a 2-step process. For example, the policy measures’ impact on the annual probability of diagnosis will be considered (Figure 4). The quantification of the policy measures’ impact on the remaining steps in the cure cascade followed equivalent steps.

In the first step, an expert panel was asked to address 2 important questions:

1. In the absence of any policy measure related to diagnostics, what would be the expected annual percentage of individuals infected with HCV getting diagnosed (P_D,Min_)?
2. With all political measures related to diagnostics fully operational, what would be the expected annual percentage of individuals infected with HCV getting diagnosed (P_D,Max_)?

The difference between these 2 values defines the maximum impact (△_Max_) the policy measures can have on the annual probability of diagnosis (Figure 4).

In the second step, each policy measure’s contribution to the maximum impact was determined. A simple solution could be to assume that this amount is equally distributed across the policies in question. In the case of 10 policy measures impacting diagnostic rates, this would mean that each policy measure’s implementation would add one-tenth of △_Max_ to the annual rate of diagnosis starting at P_D,Min_. However, it is highly unlikely that policy measures related to patient advocacy will have the same impact on annual diagnosis rates as those related to general population screening.

Conjoint analysis techniques were used to determine a more realistic relative weight of each policy measure (and their level of implementation) on the maximum possible impact. These techniques are ideally suited to quantify the relative importance of different, often qualitative, attributes considered important for decision-making. The same expert panel that was requested to pronounce itself on the maximum impact of the policy measures on the annual probability of diagnosis was consulted to determine these relative weights using the commercial ACA Sawtooth Software (Figure 4) [36].

It is important to note that the global policy impact defined for the LEHC project is solely a temporary solution based on an average value, as it is thought that each policy will have an inherent impact that may differ from those of the others. The found solution was based on a qualitative approach resulting from a consensus among hepatitis C experts to fill the gap of no quantitative studies being found on each of the 24 PHPs considered for the LEHC model. Considering this, further research is necessary to identify the impacts of each of the studied PHP, which requires various research lines encompassing elements such as different cultures and legislations in different countries. Nonetheless, the developed work in the LEHC project provides a basis for discussion toward future research, having defined a maximum policy impact of 30% in the model. Although defining the global impact of PHP is not within this study’s scope, it is still important to identify it as a possible limitation.

#### Model Initialization

On the basis of the 2 main Markov model components of the LEHC health policy tool (CHC disease progression and CHC cure cascade), all individuals of a particular population subgroup (further stratified by age and gender) can be represented in a set of mutually exclusive states through which they move over time.

Starting from the current (ie, 2017) configuration of individuals over the different model states, the health policy tool aims to assess the viability of eliminating CHC infection by 2030, under different scenarios of policy measure implementation, in the different countries and population subgroups being assessed. Elimination is defined as a percentage reduction in CHC prevalence between 2015 and 2030 above a certain high threshold (eg, 90%).

Given the complexity of the model, considering simultaneously up to 9 progression health states, 8 cure cascade states, 7 age groups, and 2 genders, it is easily understood that the population is stratified over up to 1000 mutually exclusive model states at each point in time. Knowing that a large part of the population with CHC is suspected to be undiagnosed in many countries, it is highly unlikely to find any country or population subgroup where an exact or approximate distribution of all individuals over these many states might be available.

An informal back-calculation [37] approach was used to infer a plausible current configuration of individuals over the different model states. To this end, the best possible qualitative, quantitative, longitudinal, cross-sectional, and historical data available for each population subgroup were used. On the basis of these data, the informal back-calculation involved choosing parameter values (such as incidence and diagnosis rates), observing how closely the model simulations for the most recent years are aligned with the scarce data available, and adjusting accordingly.

#### Population Groups

The model integrates 5 different populations (Table 2) that are susceptible to HCV infection. These populations are dynamic inside each group but are considered mutually exclusive.

**Table 2:** Population groups included in the epidemiological model.

Group	Definition
Remaining population	This population includes the country’s overall population, excluding all the individuals from the remaining population groups (people who inject drugs, prisoners, and people who use blood products).
People who inject drugs	This population represents the national population of injectable drug users. Note that this population does not include drug users who do not inject drugs. As there is a significant overlap between people who inject drugs and prisoners, as a great number of prisoners are also people who inject drugs, and as it has been assumed that the different groups in the model are mutually exclusive, it was defined that this population group only includes the people who inject drugs who are not incarcerated.
Prisoners	This population includes convicted prisoners with sentences >1 year. As the model was constructed on an annual basis, all the preventive prisoners and convicted prisoners with penalties <1 year were excluded.
People who receive blood products	This population includes individuals who have received blood transfusions or blood derivate products.
Individuals infected through vertical transmission	This population is included within the remaining population group. It represents all individuals who were infected through mother-to-child transmission during birth.
Total population	This population is composed of the whole country’s population, meaning that it is the sum of all other population groups.

To simulate the epidemiological evolution of hepatitis C for the retrospective period between 1950 and 2016 (model initialization), a series of prespecified inputs must be collected from the available literature for each of the different population groups. Some of these inputs are also required prospectively for the period between 2017 and 2030. For all the missing information, a series of assumptions must be made. The methodology for data gathering, followed for data processing, is described below.

#### Demography

Demography refers to the population size over the years. For the remaining population, it consists of the resident population size of a country and the number of live births to estimate the changes in population size over time, every year between 1950 and 2018, stratified by gender and age classes.

The other population groups consist of the total number of individuals belonging to each one per year from 1950 to 2030 stratified by gender and age class.

#### Turnover

Turnover refers to the number of people who enter (turnover in) or exit (turnover out) the population group each year per age group between 1950 and 2030.

For the remaining population, turnover in consists only of live births. Turnover out should be equal to the number of people who enter other population groups.

For people who inject drugs, turnover in consists of people who start injecting drugs each year. By contrast, turnover out consists of all individuals who stop injecting drugs each year, thus leaving this population and becoming part of the remaining population.

For prisoners, turnover in consists of arrested and incarcerated people during that year, whereas turnover out consists of all individuals released from prison during that year, thus becoming part of the remaining population.

For patients receiving blood products, turnover in consists of people who enter the population each year. As people never stop being ex-transfused, it was assumed that there is no turnover out for this population other than mortality, which is a structural component of the model.

#### Background Mortality

Background mortality refers to the annual probability of death in each population group per year between 1950 and 2030 stratified by age group and gender.

#### Hepatitis C Incidence

Hepatitis C incidence refers to the number of new chronic HCV infections in each population group per year between 1950 and 2018 stratified by gender and age class. In the remaining population, all new infections occurring in the other population groups should be accounted for.

#### Hepatitis C Prevalence

Hepatitis C prevalence in the remaining population refers to the predicted prevalence of chronic HCV infections over the years, stratified by gender and age class. Only patients with CHC, who do not belong to any of the other population groups should be considered.

Hepatitis C prevalence in the other population groups refers to the total number of chronic HCV infections in each population per year between 1950 and 2018 stratified by gender and age class.

#### Diagnosis

Diagnosis refers to the annual probability of an infected individual being diagnosed in each population group per year between 1950 and 2018 according to gender, age group, and disease stage.

#### Retain in Care

Retain in care refers to the annual probability of a diagnosed individual being retained in care in each population group per year between 1950 and 2018 according to gender, age class, and disease stage.

#### Treatment 1

Treatment 1 refers to the annual probability of an infected individual being treated for the first time against hepatitis C if already retained in care in each population group between 1950 and 2018 according to gender, age group, and disease stage.

#### Treatment 2

Treatment 2 represents the patients’ second treatment against CHC and should only consider DAA class therapies. It refers to the annual probability of an infected individual being retreated if retained in care and previously treated in each population group according to age and disease stage for the years between 1950 and 2018.

#### SVR in Treatment 1

SVR in treatment 1 represents the probability of a patient achieving an SVR in the first treatment against HCV in each population group for the period between 1950 and 2030.

#### SVR in Treatment 2

SVR in treatment 2 represents the probability of a patient achieving an SVR with the retreatment with DAA therapy (treatment 2) in each population group for the period between 1950 and 2030.

### Ethical Considerations

In compliance with Portuguese legislation, our study did not involve access to patients’ clinical data, personal interactions with them, individual medical records, or consultations with hospital databases. Our research solely relied on elements published in peer-reviewed scientific literature or official state reports. As a result, ethical committee approval was not necessary.

## Results

The construction of the LEHC model had to proceed with an adaptation to the practice, involving the construction of proxies in the modeling process. The LEHC model comprised an average production time of 3000 hours per country, considering all the interventions, from data collection to obtaining a fitting.

The model was applied to a set of countries; studies in 5 of these countries have already been completed or are currently under publication process [38-42], and those in the remaining countries are in different conclusion phases, with data being available on the LEHC website for the period between 2017 and 2019.

The double imputation model allowed the assessment of a set of indicators such as LT, incidence, and deaths year by year until 2030 in different risk groups.

Initial feedback from decision makers in pilot countries suggests that the modeling tool was helpful for the politicians. The tool allows people to simulate even seemingly strange or counterintuitive solutions with privacy simply to verify data movements’ sensitivity. The simulation processes enabled the health decision makers and the politicians to provide answers about the tool’s performance limits and illustrate with concrete, measurable, and externally verifiable facts [38-42].

## Discussion

### Principal Findings

It was possible to create a gamified system in which even people who are not experts in the field of hepatitis C can have an informed opinion and simulate different technical solutions for their country. This technological opacity solution allows people to use a modeling tool without the need for knowledge about medicine or mathematics. The gamification of the tool allows people to get an idea of which policies can be invoked and the corresponding degree of application by testing different scenarios. This process enables people to be knowledgeable about the impact of policies on different populations and the country as a whole.

The “democratization” provided by gamification puts citizens involved in patient advocacy on the same level as people linked to patient associations and other civil society members (patients, family members, and employers, among others). This is due to allowing the discussion with a technical basis regarding the impact of policies that can be implemented and the practical limitations in contexts with a strong will to eliminate hepatitis C by 2030.

Similarly, for politicians and health system decision makers, the gamification of scenarios allows an instantaneous simulation of each policy’s impact on the main health indicators explained earlier.

### Limitations

Several types of limitations were identified throughout LEHC, and some of them are highlighted subsequently.

#### Model Construction

In terms of model construction, there were many requests from politicians and health system decision makers to add a component of financial analysis to the routines and simulation algorithms, which is not very complex. However, it was not possible to take this step because of the lack of funding for this component. This would also further increase the number of statistical series to be collected and worked on, which would not be feasible with the available resources. Nevertheless, we consider that this new functionality would be very advantageous, as it would allow countries to simulate the policy itself and verify the financial impact that one option has in comparison with another, although it may only be in order of magnitude.

How countries collect and systematize their data varies enormously, complicating and sometimes even preventing direct comparability between identical statistical series. In addition, there is still a need to unify the systematics and ontologies related to hepatitis C (at least at the European level). Even at the country level, the existence of autonomous regions means that there are different health conditions, health legislations, and assistance rights of citizens existing in parallel, making it difficult to obtain a national framework. The solution we found was to work with only 1 region, for example, as was the case in the United Kingdom, where LEHC was applied to England (study on which is almost complete but not finalized), or to work in all autonomous regions, as was the case in Spain, by creating unification proxies, which required tremendous effort and caused great difficulty. In our reading, the only solution for the future will be to create an LEHC at 2 levels, national and regional, for countries that have autonomous health systems. If this option is to be chosen, the efforts made for a country should be multiplied by the number of autonomous regions in that country, provided that all the regions have detailed data for hepatitis C.

#### Model Application

It must be noted that no country managed to have all the necessary data for the LEHC model. When the series existed in >1 bibliographic source, they were almost always partial or contradictory. There was a need to produce hundreds of proxies with the support of local research teams and validate the data with the NABs based on international experts’ experiences.

Even the statistical series available on hepatitis C and populations with risk behaviors for this disease have always been incomplete in terms of the model’s requirements. Specific data often focused on specific populations. Even when considering a population with risk behaviors, data sometimes represented specific time periods, specific regions, or different data collection methodologies, which necessitated enormous efforts in finding common denominators.

Another limitation of the model was that the experts constituting the NABs often seemed to be comfortable assessing the impact of some of the modeled policies but not the others. An important predictor of this difficulty was the professional experience of NAB members with specific populations.

### Comparison With Prior Work

This work differs from the existing models, as it targets the simulation of PHPs focused on hepatitis C in an innovative way by using gamification processes, allowing citizens to simulate PHP application in the disease, a possibility that until now has been reserved for specialists. The epidemiological modeling in LEHC was seamlessly integrated into decision-making processes through a unified solution.

As in previous works mentioned in the literature review [15,18-21,23], modeling allowed obtaining a set of indicators, as explained above. Similarly, as stated in the appointed application articles for this model [38-42], although there were differences in the year of elimination of hepatitis C (according to WHO criteria), these were not structural until the COVID-19 pandemic. It must be noted that COVID-19 represents a threat to allocated efforts to achieve the elimination of hepatitis C by 2030, with the disease elimination programs being highly impacted [43-45]. In addition, the results that were different were essentially because of distinct assumptions regarding the implementation intensity of PHPs, which points toward an interesting concurrent validity.

### Conclusions

The LEHC model combines epidemiological mathematical modeling in its classic sense with the modeling of political decision-making in a single solution. It can simulate different policies with different implementation degrees, thus allowing the anticipation of results concerning different options, making it a potential tool for political decision-making on hepatitis C.

One of the main conclusions is that the tool is able to generate solutions that can be specific for each reality, in which each country ends up needing a set of specific PHPs to address the need to eliminate hepatitis C by 2030. This means that there is an enormous cultural component in the sense of diverse lifestyles, legislations, and social history in general, which the LEHC tool, with its adaptive flexibility, responds to.

This does not mean that the values provided by the LEHC tool should be understood as exact numbers from the past or the future. Like all models, LEHC depends heavily on the quality of the input data, and it was very clear to the authors that there is unlimited dominance in the data from all countries, namely for highly specific data, such as information about people who inject drugs. Therefore, this explains how even the largest organizations have made epidemiological corrections of 40% to 50% in recent years. In our reading, this limitation does not remove the merit and interest of the LEHC tool, and on the contrary, it encourages research and may also be a call for research.

The general meaning of PHP turns out to almost always be the intensity of its use and the potential consequences that can be gained by intensifying it. This is where LEHC provides invaluable help to decision makers and patient advocates, as per feedback that has been collected from these 2 social groups.

The integration of the tool into a gamification system allows anyone interested in hepatitis C to forecast both the current policies that appear as standard and those that are intended to be tested to seek alternative paths. Therefore, it is legitimate to point LEHC as a patient advocacy tool.

Hepatitis C is going through a very problematic phase, discovering millions of cases that still exist outside the health systems; those that were in the area of knowledge in general have already been integrated into the processes of diagnosis and treatment. Because those who remain outside the system are socially opaque, they cannot not be integrated into treatment using just the usual clinical and public health measures because if that were possible, after 8 years of efforts by the health systems, these people would already have been treated.

We have to get out of the standard practices and find social forms based on social segmentation, with PHPs that cover beyond the usual spheres of health to capture the tens of millions of people who are outside. If such a strategy is not adopted, we will somehow be doomed to have viral reservoirs in uncertain parts of the social system, which will feed the reinfections of those who have already been treated as well as disseminators among those who are not yet sick.

LEHC being an integrated and articulated structure of PHPs can make a very positive contribution to join all the other structures that are currently being developed throughout the disease cure cascade.

## Abbreviations

ACA: adaptive conjoint analysis
CHC: chronic hepatitis C
DAA: direct-acting antiviral
DC: decompensated cirrhosis
HCC: hepatocellular carcinoma
HCV: hepatitis C virus
LEHC: Let’s End HepC
LT: liver transplant
NAB: National Advisory Board
PHP: public health policy
PLT: post–liver transplant stage
SVR: sustained virologic response
WHO: World Health Organization
